# Supplementary material for: Directed propulsion of spherical particles along three dimensional helical trajectories
Source: Nat Commun. 2019 Jun 12;10:2575. doi: 10.1038/s41467-019-10579-1 (PMC6561940; doi:10.1038/s41467-019-10579-1)
Supplement: Supplementary file 1 — Supplementary Information [file 41467_2019_10579_MOESM1_ESM.pdf]

Directed propulsion of spherical particles along three dimensional helical trajectories.

Lee *et al.*

Supplementary Information

Directed propulsion of spherical particles along three dimensional helical trajectories

Jin Gyun Lee[1], Allan M. Brooks[2], William A. Shelton[1], Kyle J. M. Bishop[3], and Bhuvnesh Bharti[*1]

[1]Cain Department of Chemical Engineering, Louisiana State University, Baton Rouge, LA 70803
[2]Department of Chemical Engineering, Pennsylvania State University, University Park, PA 16802
[3]Department of Chemical Engineering, Columbia University, New York, NY 10027

[*]Address correspondence to bbharti@lsu.edu

Supplementary Figures

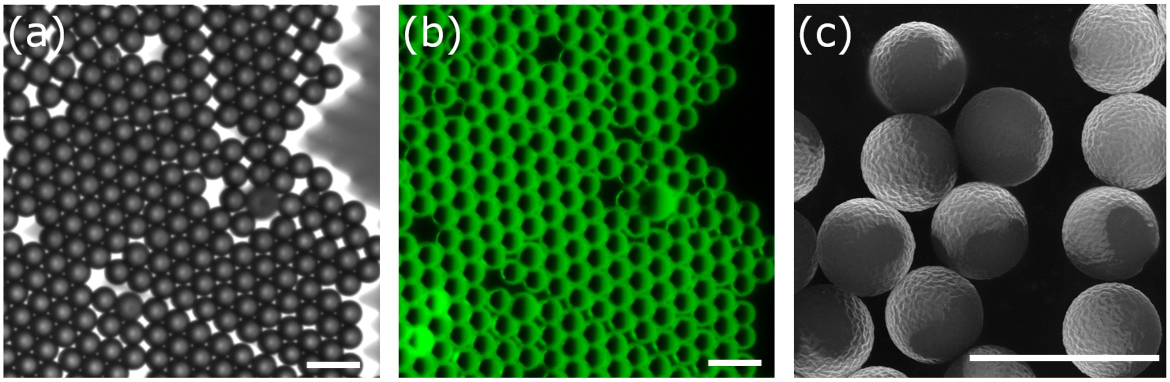

Supplementary Figure 1: (a-b) Bright field and fluorescent micrographs of polystyrene microspheres coated with triangular gold patch. In (b) the patch is the dark region on the green microspheres. (c) Scanning electron micrograph of the microspheres with triangular gold patch (light grey). Scale bar in (a-c) is 10 μm.

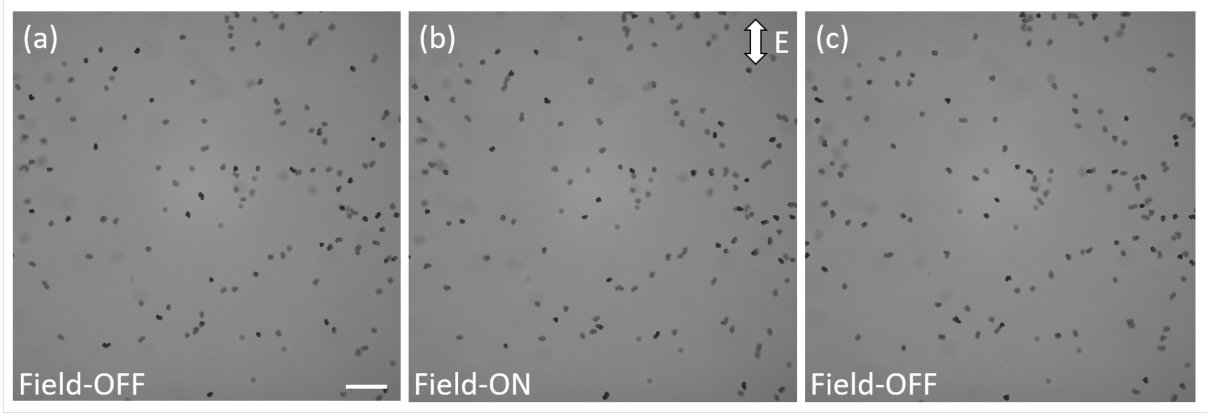

Supplementary Figure 2: Overlay of microscope images showing the movement of non-patchy negatively charged particles (radius 1.0 μm) in AC-electric field (a) OFF, (b) ON (150 V/cm, 10 kHz), and (c) OFF state. Each image is an overlay of 27 frames accumulated in 5s, and shows the trajectory of the particle in the field OFF$\rightarrow$ON$\rightarrow$OFF state. Scale bar in (a) is 50 μm.

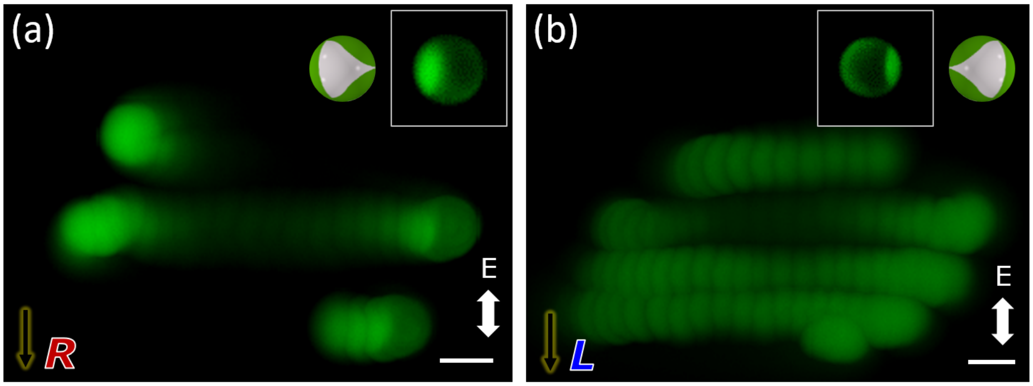

Supplementary Figure 3: Fluorescence microscope images showing the motion of one particle along two different helical trajectories of opposite handedness. Here the radius of the particle is 2.6 μm, and metal vapor deposition angle is $\varphi = 10°$. The magnitude and frequency of the applied field are 195 V/cm and 10 kHz. Scale bars in (a) and (b) are 5 μm.

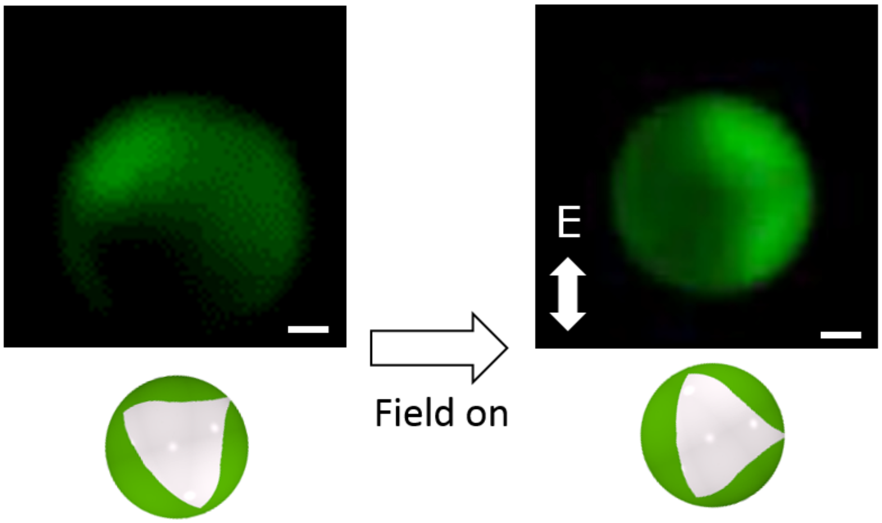

Supplementary Figure 4: Images showing the rapid alignment of a particle upon application of the external field (150 V/cm, 10 kHz).The particle radius is 2.5 μm, and the deposition angle is $\varphi = 10°$. Scale bars are 1 μm.

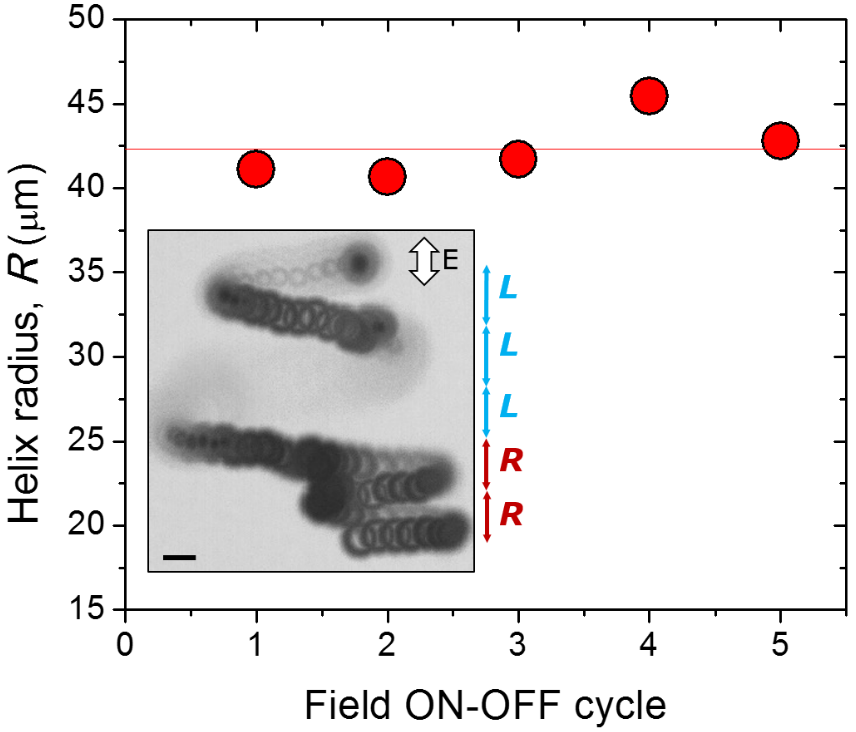

Supplementary Figure 5: Fitted radius of the helical trajectory for a particle subject to five field ON-OFF cycles (corresponding to data shown in Figure 3a). The radius is estimated by fitting the particle trajectory during the field ON state as illustrated in Figure 2a and 2b in main article. Scale bar in inset is 3 μm.

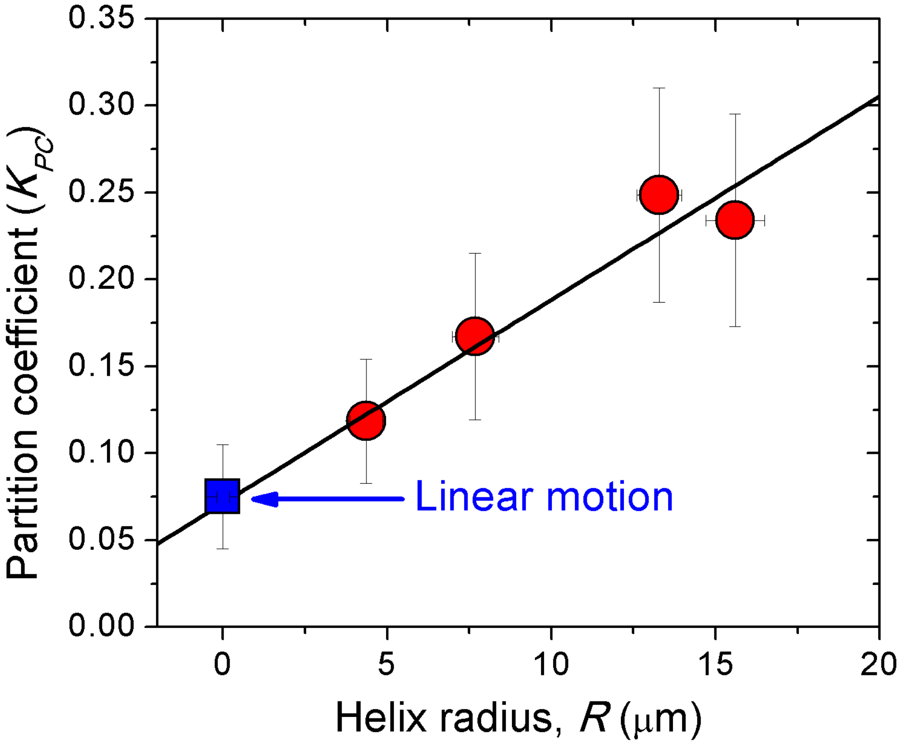

Supplementary Figure 6: The increase in partition coefficient K_{PC} at t = 15 minutes, with increasing radius of helical trajectory. The symbols are experimental data points and the straight line is a guide to the eye. The experiments were performed at E = 260 V/cm, 10 kHz on particles of radius 1 μm, and f was varied from 0.001 to 0.05. The helix radius of particle was obtained by digital analysis of brightfield microscopic overlay images of the particles tunneling through the membrane as shown in Figure 6c of main article. The number of particles in the compartments at t = 0 minute and t = 15 minutes were determined by fluorescent imaging at bottom of compartments A and B which lie in different focal planes. The square symbol represents the case of linearly propelled particles, and follows the linear decrease in K_{PC} with decreasing R. The x- and y-error bars respectively represent the standard deviation of the experimental values of partition coefficient and helix radius of the particles.

Supplementary Note 1: ICEP of particles with C_s symmetry

Here, we present a more detailed analysis of the ICEP motion of particles with one plane of mirror symmetry (C_s point group). Our analysis follows closely that of Brooks *et al.*;[1] however, the results for the C_s particle are new. We consider the motion of a polarizable particle immersed in an unbounded electrolyte and subject to an oscillating electric field, $\boldsymbol{E}_o(t) = E_o \cos(\omega t)\boldsymbol{e}_z$, with magnitude E_o and frequency ω. For sufficiently small frequencies and field strengths, the translational and rotational velocity of the particle can be expressed as

$$\boldsymbol{U} = \frac{\varepsilon a}{\eta}\boldsymbol{C} : \boldsymbol{E}_o\boldsymbol{E}_o, \tag{1}$$

$$\boldsymbol{\Omega} = \frac{\varepsilon}{\eta}\boldsymbol{D} : \boldsymbol{E}_o\boldsymbol{E}_o, \tag{2}$$

where ε and η are, respectively, the permittivity and the viscosity of the electrolyte, a is the size of the particle, and $\boldsymbol{C}$ and $\boldsymbol{D}$ are dimensionless tensors, which share the symmetry of the particle.[2] In the analysis below, we scale time by the electroviscous scale $\eta/\varepsilon E_o^2$ and length by the particle radius a.

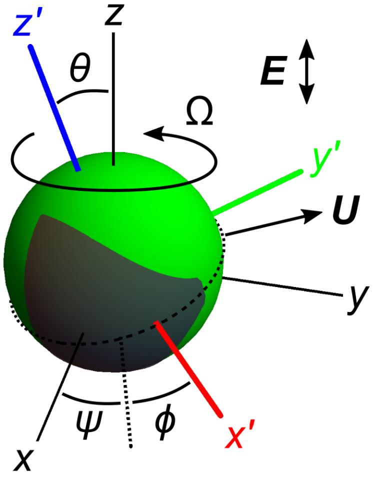

Supplementary Figure 7: Patchy particle with C_s symmetry showing the particle frame (x', y', z') and the lab frame (x, y, z). These coordinate systems can be related by the Euler angles (ψ, θ, ϕ).[3] The particle is invariant to reflections about the $x'y'$ plane. The field is applied along the z direction.

Coordinate Systems. To describe the rigid-body motion of the particle, we introduce two coordinate systems: a stationary system and a moving system, which is fixed to the particle and participates in its motion. A vector $\boldsymbol{v}'$ expressed in the moving system is related to the same vector $\boldsymbol{v}$ in the stationary system as $v'_i = R_{ij}v_j$ where $\boldsymbol{R}$ is an orthogonal rotation matrix that depends on the orientation of the particle (e.g., on the Euler angles ϕ, θ, ψ). Similarly, the components of the tensors, $\boldsymbol{C}$ and $\boldsymbol{D}$, in the stationary system are related to

those in the moving system as

$$C_{ijk} = R_{pi} R_{qj} R_{rk} C'_{pqr}, \tag{3}$$

$$D_{ijk} = R_{pi} R_{qj} R_{rk} D'_{pqr}. \tag{4}$$

The components C'_{pqr} and D'_{pqr} are independent of particle orientation and depend only on particle shape and/or patchiness. Knowledge of these constants allows for computation of the particle trajectory in accordance with equations (1) and (2) and the kinematics of rigid-body motion. In general, there are 18 quantities associated with each tensor (27 components less 9 relations of the form $C_{ijk} = C_{ikj}$ due to the fact that $E_j E_k = E_k E_j$). Particle symmetries can be used to further simplify these tensors and thereby constrain the types of accessible particle motions.

Particle Symmetry. Here, we consider particles with mirror symmetry about the $z = 0$ plane (C_s point group) as specified by the orthogonal matrix $\boldsymbol{Q}$,

$$\boldsymbol{Q} = \begin{bmatrix} 1 & 0 & 0 \\ 0 & 1 & 0 \\ 0 & 0 & -1 \end{bmatrix}. \tag{5}$$

Invariance of the particle with respect to the operation $\boldsymbol{Q}$ implies the following relationships among the components of the shape tensors

$$C'_{ijk} = Q_{ip} Q_{jq} Q_{kr} C'_{pqr}, \tag{6}$$

$$D'_{ijk} = |\boldsymbol{Q}| Q_{ip} Q_{jq} Q_{kr} D'_{pqr}. \tag{7}$$

These symmetry relations reduce the number of independent quantities needed to specify the tensors C' and D' from 18 to 10 and 8, respectively.

Rotational Dynamics. The rotation tensor in the particle frame D'_{ijk} can be expressed using eight parameters:

$$\boldsymbol{D'} = \left[\begin{pmatrix} 0 & 0 & D'_{131} \\ 0 & 0 & D'_{132} \\ D'_{131} & D'_{132} & 0 \end{pmatrix} \begin{pmatrix} 0 & 0 & D'_{231} \\ 0 & 0 & D'_{232} \\ D'_{231} & D'_{232} & 0 \end{pmatrix} \begin{pmatrix} D'_{311} & D'_{321} & 0 \\ D'_{321} & D'_{322} & 0 \\ 0 & 0 & D'_{333} \end{pmatrix} \right]. \tag{8}$$

Assuming $\boldsymbol{E}_o = E_o \boldsymbol{e}_z$, the equations governing the particle's rotational motion can be expressed using Euler angles as

$$\dot{\phi} = (a + b\cos 2\phi - c\sin 2\phi)\cos^2\theta + (g + h\cos 2\phi + k\sin 2\phi)\sin^2\theta, \tag{9}$$

$$\dot{\theta} = \tfrac{1}{2}(d + c\cos 2\phi + b\sin 2\phi)\sin 2\theta, \tag{10}$$

$$\dot{\psi} = (f - b\cos 2\phi + c\sin 2\phi)\cos\theta. \tag{11}$$

where the coefficients are related to the components of the rotation tensor as

$$\begin{array}{llll} a = D'_{333} - D'_{131} - D'_{232} & c = D'_{132} + D'_{231} & f = D'_{131} + D'_{232} & h = \tfrac{1}{2}(D'_{322} - D'_{311}) \\ b = D'_{131} - D'_{232} & d = D'_{132} - D'_{231} & g = \tfrac{1}{2}(D'_{311} + D'_{322}) & k = D'_{321} \end{array}$$

Due to rotational symmetry about the field axis, the rates of motion depend only on the angles ϕ and θ (not ψ).

Depending on the coefficients, there are three types of steady-state solutions: (1) the particle orients its mirror plane perpendicular to the field ($\theta_o = 0$), (2) the particle orients its mirror plane parallel to the field ($\theta_0 = \pi/2$), and (3) the particle orients its mirror plane oblique to the field. Here, we focus on the third scenario, corresponding to helical motions, in which the particle evolves to a stable orientation characterized by angles ϕ_o and θ_o at which $\dot{\phi} = \dot{\theta} = 0$. The angle ϕ_o satisfies the equation

$$0 = d + c\cos 2\phi_o + b\sin 2\phi_o \tag{12}$$

which admits solutions only when $b^2 + c^2 > d^2$. Similarly, the angle θ_o satisfies the equation

$$-\tan^2\theta_o = \frac{a + b\cos 2\phi_o - c\sin 2\phi_o}{g + h\cos 2\phi_o + k\sin 2\phi_o} < 0 \tag{13}$$

which admits solutions only when the right-hand-side is negative. The equation for θ_o admits four solutions on the domain $(-\pi, \pi)$. Because $\tan^2\theta$ is an even π-periodic function, a solution θ_o on the domain $(0, \pi/2)$ can be mapped to three other solutions: $-\theta_o$, $\pi - \theta_o$, and $\theta_o - \pi$. As shown below, these four stable orientations correspond to four helical trajectories of different handedness (left or right) and direction (positive or negative z-direction). The angular velocity of the helical trajectory is

$$\Omega \equiv \dot{\psi} = (f - b\cos 2\phi_o + c\sin 2\phi_o)\cos\theta_o. \tag{14}$$

The solution (12) and (13) is only stable for certain values of the rotation tensor coefficients. Linearizing equations (9) and (10) about the solution (ϕ_o, θ_o), we obtain

$$\begin{bmatrix}\dot{\phi}\\ \dot{\theta}\end{bmatrix} = \boldsymbol{J}\begin{bmatrix}\phi - \phi_o\\ \theta - \theta_o\end{bmatrix} \tag{15}$$

where $\boldsymbol{J}$ is the Jacobian matrix. Small disturbances from the fixed point (ϕ_o, θ_o) decay in time provided that the two eigenvalues of the Jacobian matrix have negative real parts; equivalently, both the trace and the determinant of the Jacobian matrix must satisfy the conditions

$$\mathrm{tr}(\boldsymbol{J}) = 2\left(d\cos^2\theta_o + (k\cos 2\phi_o - h\sin 2\phi_o)\sin^2\theta_o\right) < 0 \tag{16}$$

$$\det(\boldsymbol{J}) = \sin^2 2\theta_o(b\cos 2\phi_o - c\sin 2\phi_o)(a - g + (b+h)\cos 2\phi_o - (c+k)\sin 2\phi_o) > 0 \tag{17}$$

ICEP of C_s particles leads to stable helical trajectories provided that the coefficients of the rotation tensor satisfy the above conditions. Figure 8 shows one possible phase portrait in the $\phi\theta$-plane.

Translational Dynamics. In general, the translation tensor of a C_s particle has ten distinct coefficients:

$$\boldsymbol{C'} = \left[\begin{pmatrix} C'_{111} & C'_{121} & 0\\ C'_{121} & C'_{122} & 0\\ 0 & 0 & C'_{133}\end{pmatrix} \begin{pmatrix} C'_{211} & C'_{221} & 0\\ C'_{221} & C'_{222} & 0\\ 0 & 0 & C'_{233}\end{pmatrix} \begin{pmatrix} 0 & 0 & C'_{331}\\ 0 & 0 & C'_{332}\\ C'_{331} & C'_{332} & 0\end{pmatrix}\right]. \tag{18}$$

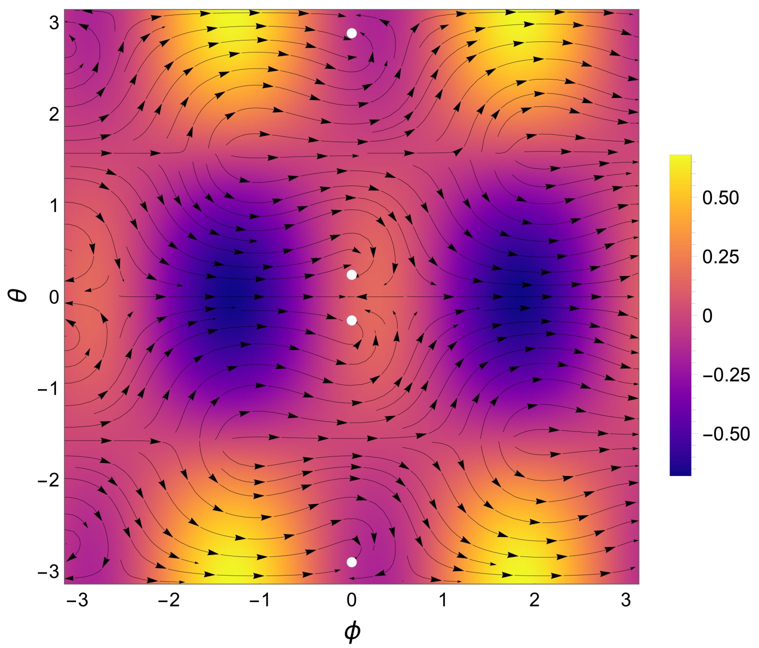

Supplementary Figure 8: Phase portrait for a C_s particle with rotation tensor coefficients $D'_{131} = -0.311$, $D'_{132} = 0$, $D'_{231} = -0.212$, $D'_{232} = 0.0516$, $D'_{311} = 0$, $D'_{321} = -0.0138$, $D'_{322} = 0.338$, $D'_{333} = 0.0812$. The colormap shows the magnitude of $\dot{\psi}$; arrows show the flow in the $\phi\theta$ plane. Stable fixed points are marked by white dots; here, the coefficients were chosen such that $\theta_o = 0.25$. The eigenvalues are $\lambda_+ = -0.1$ and $\lambda_- = -0.3$. The angular velocity at the stable fixed points is $\Omega = \pm 0.1$.

Here, the particle is oriented such that its plane of mirror symmetry is perpendicular to the z'-axis. When there exists a stable orientation in the field (ϕ_o, θ_o) satisfying equations (12) and (13), it is convenient to rotate the particle about the z'-axis such that the angle ϕ_o is identically zero. In this frame of reference, the linear velocity of the particle is given by

$$\dot{x} = V \cos \Omega t, \tag{19}$$
$$\dot{y} = V \sin \Omega t, \tag{20}$$
$$\dot{z} = U, \tag{21}$$

where the linear velocity U and the orbital velocity V are given by

$$U = C'_{222} \sin^3 \theta_o + (C'_{233} + 2C'_{332}) \sin \theta_o \cos^2 \theta_o, \tag{22}$$
$$V^2 = (C'_{133} \cos^2 \theta_o + C'_{122} \sin^2 \theta_o)^2 + \left((C'_{222} - 2C'_{332}) \sin^2 \theta_o \cos \theta_o + C'_{233} \cos^3 \theta_o\right)^2. \tag{23}$$

These dynamics trace a helical trajectory of radius $R = V/\Omega$ and pitch $P = 2\pi U/\Omega$, which depend only on the shape and/or patchiness of the particle as characterized by the tensors $\boldsymbol{C}$ and $\boldsymbol{D}$. The velocities Ω and U satisfy the following relations

$$\Omega(-\theta_o) = \Omega(\theta_o) \qquad \Omega(\pi - \theta_o) = -\Omega(\theta_o) \qquad \Omega(\theta_o - \pi) = -\Omega(\theta_o) \tag{24}$$
$$U(-\theta_o) = -U(\theta_o) \qquad U(\pi - \theta_o) = U(\theta_o) \qquad U(\theta_o - \pi) = -U(\theta_o) \tag{25}$$

If there exist a solution θ_o on the domain $(0, \pi/2)$ which corresponds to an upward-moving, right-handed helix ($U > 0$ and $\Omega > 0$), then there are three other solutions: (1) $\theta = -\theta_o$ gives a downward-moving, left-handed helix ($U < 0$ and $\Omega > 0$), (2) $\theta = \pi - \theta_o$ gives a upward-moving, left-handed helix ($U > 0$ and $\Omega < 0$), and (3) $\theta = \theta_o - \pi$ gives a downward-moving, right-handed helix ($U < 0$ and $\Omega < 0$). Each of these helices has the same radius and pitch; the selection of one or another is determined by the initial orientation of the particle in the field.

Supplementary Note 2: ICEP of patchy spheres ($\kappa a \gg 1$)

Thin Double-Layers

When the Debye screening length κ^{-1} is much smaller than the particle radius a, metalodielectric spheres are prohibited from rotating about the axis of the applied field and are therefore incapable of performing helical motions. This conclusion is supported by analysis of the following model due to Squires & Bazant[4], which applies to particles with thin double layers ($\kappa a \gg 1$) subject to weak electric fields ($aE_o \ll k_B T/e$). Moreover, we assume that the applied frequency ω is small relative to the "RC time" of the particle—that is, $\omega a/\kappa D \ll 1$, where D is the ion diffusivity.[5] Under these conditions, we can neglect temporal variations in the ion distributions, which rapidly approach steady state.

Within the electrolyte surrounding the particle, the electric potential is governed by the Laplace equation for steady-state Ohmic conduction

$$\nabla^2 \Phi = 0 \quad \text{(in the electrolyte)} \tag{26}$$

Far from the sphere, we assume that the field is uniform

$$-\nabla \Phi(\boldsymbol{r}) = \boldsymbol{E}_o \quad \text{for} \quad r \to \infty \tag{27}$$

where $r^2 = \boldsymbol{r} \cdot \boldsymbol{r}$. At the surface of the sphere, the ionic current normal to the particle surface vanishes such that

$$\boldsymbol{n} \cdot \nabla \Phi = 0 \quad \text{for} \quad r = a \tag{28}$$

where $\boldsymbol{n}$ is the unit normal vector directed out from the sphere of radius a. The resulting potential Φ and electric field $-\nabla\Phi$ are given by

$$\Phi(\boldsymbol{r}) = -\boldsymbol{E}_o \cdot \boldsymbol{r} \left(1 + \frac{a^3}{2r^3}\right) \tag{29}$$

$$-\nabla \Phi(\boldsymbol{r}) = \boldsymbol{E}_o - \frac{a^3}{2r^3}\left(3(\boldsymbol{E}_o \cdot \hat{\boldsymbol{r}})\hat{\boldsymbol{r}} - \boldsymbol{E}_o\right) \tag{30}$$

where $\hat{\boldsymbol{r}} = \boldsymbol{r}/r$ is the unit vector in the r-direction.

At the surface of the sphere, the field acts on the field-induced surface charge to produce a finite slip velocity tangent to the surface

$$\boldsymbol{u}_s(\boldsymbol{r}) = \frac{\varepsilon}{\eta} \zeta(\boldsymbol{r}) \nabla \Phi(\boldsymbol{r}) \quad \text{for} \quad r = a \tag{31}$$

where the zeta potential is the surface potential $\Phi_s(\boldsymbol{r})$ less the potential $\Phi(\boldsymbol{r})$ outside the double-layer, $\zeta(\boldsymbol{r}) = \Phi_s(\boldsymbol{r}) - \Phi(\boldsymbol{r})$. The linear and angular velocities of the sphere are given by surface integrals of the slip velocity[4,6]

$$\boldsymbol{U} = -\frac{1}{4\pi a^2} \int_{\mathcal{S}_p} \boldsymbol{u}_s(\boldsymbol{r}) \mathrm{d}\mathcal{S} \tag{32}$$

$$\boldsymbol{\Omega} = -\frac{3}{8\pi a^4} \int_{\mathcal{S}_p} \boldsymbol{r} \times \boldsymbol{u}_s(\boldsymbol{r}) \mathrm{d}\mathcal{S} \tag{33}$$

These velocities are equivalent to those of equations (1) and (2).

Importantly, helical motions require particle rotations about the axis of the field such that $\boldsymbol{E}_o \cdot \boldsymbol{\Omega} \neq 0$; however, the above model implies that $\boldsymbol{E}_o \cdot \boldsymbol{\Omega} = 0$ regardless of the field induced zeta potential $\zeta(\boldsymbol{r})$. To show this, consider that

$$\boldsymbol{E}_o \cdot \boldsymbol{\Omega} = -\frac{3\varepsilon}{8\pi\eta a^4} \int_{\mathcal{S}_p} \zeta(\boldsymbol{r}) \boldsymbol{E}_o \cdot (\boldsymbol{r} \times \nabla\Phi(\boldsymbol{r})) \mathrm{d}\mathcal{S} = 0 \tag{34}$$

where the quantity $\boldsymbol{E}_o \cdot (\boldsymbol{r} \times \nabla\Phi(\boldsymbol{r}))$ in the integrand is identically zero at every point on the surface. We therefore conclude that helical motions are not possible for spherical particles with thin double layers. By contrast, the above model is perfectly capable of describing the translation of patchy spheres as well as the rotation of non-spherical particles.

Supplementary Note 3: ICEP of patchy spheres ($\kappa a \sim 1$)

In this Note, we present a more general model for the induced charge electrophoresis (ICEP) of patchy spheres in an unbounded fluid subject to a homogeneous electric field $\boldsymbol{E}_o$. The model is based on the Poisson-Nernst-Planck-Stokes system for ion transport and fluid flow, in which the electric potential Φ is governed by the Poisson equation, the ion concentration $c_\pm$ by the Nernst-Planck equation, and the fluid velocity $\boldsymbol{u}$ and pressure p by the Stokes equations. This model accounts for the finite thickness of the electric double-layer and is solved numerically to determine the shape tensors $\boldsymbol{C}$ and $\boldsymbol{D}$ that characterize particle motion in the field. We again assume that the applied field is weak ($eaE_o/k_BT \ll 1$) and slow ($\omega a/\kappa D \ll 1$); however, these conditions may be violated in experiments. We leave the treatment of stronger and/or faster fields for future work.

Model Formulation

Ion Transport

The spherical particle is surrounded by a symmetric monovalent electrolyte of bulk concentration c_o. The concentrations $c_\pm$ of positive/negative ions in solution are described by the Nernst-Planck equation

$$\nabla \cdot \boldsymbol{j}_\pm = \nabla \cdot \left(-D_\pm \nabla c_\pm \pm \frac{eD_\pm}{k_B T} c_\pm \boldsymbol{E} \right) = 0 \tag{35}$$

where $\boldsymbol{j}_\pm$ is the flux of positive/negative ions. The flux includes contributions due to ion diffusion with diffusivity $D_\pm$ and migration in the electric field $\boldsymbol{E} = -\nabla\Phi$. Transport due to fluid convection is negligible by comparison and therefore omitted. For simplicity, we assume a common diffusivity for positive and negative ions such that $D_+ = D_- = D$. The electric potential Φ within the electrolyte is described by the Poisson equation

$$\nabla^2 \Phi = -\frac{\rho_e}{\varepsilon} \quad \text{with} \quad \rho_e = e(c_+ - c_-) \tag{36}$$

where ρ_e is the charge density.

The above equations are subject to boundary conditions at the surface of the spherical particle ($r = a$) and far from the particle ($r \to \infty$). Far from the sphere, the concentration approaches its bulk value, and the potential approaches that of the applied field $\boldsymbol{E}_o$

$$c_\pm(\boldsymbol{r}) = c_o \quad \text{for} \quad r \to \infty \tag{37}$$

$$\Phi(\boldsymbol{r}) = -\boldsymbol{r} \cdot \boldsymbol{E}_o \tag{38}$$

At the surface of the sphere, there is no flux of ions normal to the particle surface

$$\boldsymbol{n} \cdot \boldsymbol{j}_\pm(\boldsymbol{r}) = 0 \quad \text{for} \quad r = a \tag{39}$$

The electric potential is subject to the following conditions on and off the metal patch

$$\Phi(\boldsymbol{r}) = \zeta + \boldsymbol{\gamma} \cdot \boldsymbol{E}_o \qquad \text{for } r = a \text{ on the patch} \tag{40}$$

$$-\varepsilon \boldsymbol{n} \cdot \nabla \Phi(\boldsymbol{r}) = \sigma \qquad \text{for } r = a \text{ off the patch} \tag{41}$$

where ζ is the surface potential on the patch in the absence of the field, and σ is the surface charge density off the patch (independent of the field). The vector $\boldsymbol{\gamma}$ describes how the potential on the patch changes in the field, so as to maintain a state of constant (equilibrium) charge

$$q_{\text{eq}} = \int_{\mathcal{S}_p} \boldsymbol{n} \cdot (-\varepsilon \nabla \Phi(\boldsymbol{r})) \mathrm{d}\mathcal{S} \tag{42}$$

which depends on the particle surface parameters ζ and σ but not on the applied field $\boldsymbol{E}_o$. The condition (41) implies that the capacitance inside the sphere is negligible compared to that outside the sphere (i.e., the permittivity of the sphere is small: $\varepsilon_s \ll \varepsilon$). With this simplification, we need not compute the potential inside the dielectric sphere.

The above equations can be solved to determine the charge density ρ_e and the electric field $\boldsymbol{E}$ surrounding the particle. The product of these quantities is the electric force density, which drives hydrodynamic flows and thereby particle motions (see below). In the far field, the field-induced polarization of the particle and its double layer create a dipolar disturbance to the electric potential

$$\Phi(\boldsymbol{r}) = -\boldsymbol{r} \cdot \boldsymbol{E}_o + \frac{\boldsymbol{p} \cdot \boldsymbol{r}}{4\pi\varepsilon r^3} + O(r^{-3}) \tag{43}$$

The dipole moment $\boldsymbol{p}$ is related to the following integral over a bounding surface $\mathcal{S}_\infty$ at $r \to \infty$ as

$$\boldsymbol{p} = -\varepsilon \int_{\mathcal{S}_\infty} \left[\boldsymbol{n}\Phi - \boldsymbol{r}(\boldsymbol{n} \cdot \nabla\Phi) \right] \mathrm{d}\mathcal{S} \tag{44}$$

where the unit normal vector $\boldsymbol{n}$ is directed into the volume enclosed by the surface ($\boldsymbol{n} = -\hat{\boldsymbol{r}}$).

Hydrodynamics

The fluid velocity $\boldsymbol{u}$ and pressure p surrounding the spherical particle are governed by the Stokes equations for low-Reynolds number flow

$$\nabla \cdot \boldsymbol{u} = 0 \quad \text{and} \quad 0 = -\nabla p + \eta \nabla^2 \boldsymbol{u} + \rho_e \boldsymbol{E} \tag{45}$$

where η is the fluid viscosity, ρ_e is the electric charge density, and $\boldsymbol{E}$ is the electric field. Far from the sphere, the fluid is stationary. At the particle surface, the no-slip condition requires that the fluid move at the same velocity as the particle. These boundary conditions imply that

$$\boldsymbol{u}(\boldsymbol{r}) = \boldsymbol{U} + \boldsymbol{\Omega} \times \boldsymbol{r} \quad \text{for} \quad r = a \tag{46}$$

$$\boldsymbol{u}(\boldsymbol{r}) = 0 \quad \text{and} \quad p(\boldsymbol{r}) = 0 \quad \text{for} \quad r \to \infty \tag{47}$$

The particle's linear velocity $\boldsymbol{U}$ and angular velocity $\boldsymbol{\Omega}$ are determined by the conditions that the net force and torque on the particle (including both electrical and hydrodynamic contributions) are zero

$$\int_{\mathcal{S}_p} (\boldsymbol{\sigma} + \boldsymbol{\sigma}_e) \cdot \boldsymbol{n} \mathrm{d}\mathcal{S} = 0 \tag{48}$$

$$\int_{\mathcal{S}_p} \boldsymbol{r} \times (\boldsymbol{\sigma} + \boldsymbol{\sigma}_e) \cdot \boldsymbol{n} \mathrm{d}\mathcal{S} = 0 \tag{49}$$

where $\boldsymbol{n}$ is the unit normal vector directed out from the particle surface $\mathcal{S}_p$, $\boldsymbol{\sigma} = -p\boldsymbol{I} + \eta(\nabla\boldsymbol{u} + \nabla\boldsymbol{u}^T)$ is the hydrodynamic stress tensor, and $\boldsymbol{\sigma}_e = \varepsilon(\boldsymbol{E}\boldsymbol{E} - \frac{1}{2}E^2)$ is the Maxwell stress tensor.

Using the Lorentz reciprocal theorem (see derivation below), the particle velocity $\boldsymbol{U}$ and $\boldsymbol{\Omega}$ can be expressed by the following integrals of the electric force density over the volume $\mathcal{V}$ surrounding the sphere

$$6\pi\eta a\boldsymbol{U} = \int_{\mathcal{V}} (\boldsymbol{A} - \boldsymbol{I}) \cdot (\rho_e \boldsymbol{E}) \mathrm{d}\mathcal{V} \tag{50}$$

$$8\pi\eta a^3\boldsymbol{\Omega} = \boldsymbol{p} \times \boldsymbol{E}_o + \int_{\mathcal{V}} (\boldsymbol{b} - \boldsymbol{r}) \times (\rho_e \boldsymbol{E}) \mathrm{d}\mathcal{V} \tag{51}$$

Here, the tensor $\boldsymbol{A}$ and vector $\boldsymbol{b}$ are given by

$$\boldsymbol{A} = \frac{3a}{4r}(\boldsymbol{I} + \hat{\boldsymbol{r}}\hat{\boldsymbol{r}}) + \frac{3a^3}{4r^3}\left(\tfrac{1}{3}\boldsymbol{I} - \hat{\boldsymbol{r}}\hat{\boldsymbol{r}}\right) \quad \text{and} \quad \boldsymbol{b} = \frac{a^3}{r^3}\boldsymbol{r} \tag{52}$$

with $\hat{\boldsymbol{r}} = \boldsymbol{r}/r$. The contribution $\boldsymbol{p} \times \boldsymbol{E}_o$ in equation (51) describes the electric torque on the particle's net dipole moment $\boldsymbol{p}$ in the applied field $\boldsymbol{E}_o$. Given the numerical solution to the ion transport problem, we can use equations (50) and (51) to evaluate the ICEP velocity $\boldsymbol{U}$ and $\boldsymbol{\Omega}$.

Derivation of equations (50) and (51). The Lorentz reciprocal theorem[7] relates two Stokes flows $(\boldsymbol{u}, \boldsymbol{\sigma})$ and $(\boldsymbol{u}', \boldsymbol{\sigma}')$ on a common domain $\mathcal{V}$ as

$$\int_{\mathcal{S}} [\boldsymbol{u}' \cdot (\boldsymbol{\sigma} \cdot \boldsymbol{n}) - \boldsymbol{u} \cdot (\boldsymbol{\sigma}' \cdot \boldsymbol{n})] \mathrm{d}\mathcal{S} = \int_{\mathcal{V}} [\boldsymbol{u} \cdot (\nabla \cdot \boldsymbol{\sigma}') - \boldsymbol{u}' \cdot (\nabla \cdot \boldsymbol{\sigma})] \mathrm{d}\mathcal{V} \tag{53}$$

Here, the fluid volume $\mathcal{V}$ is enclosed by the particle surface $\mathcal{S}_p$ at $r = a$ and by a second spherical surface $\mathcal{S}_\infty$ at $r \to \infty$; the unit normal vector $\boldsymbol{n}$ is directed into this volume. The flow $(\boldsymbol{u}, \boldsymbol{\sigma})$ refers to the ICEP flow; the auxiliary Stokes flow $(\boldsymbol{u}', \boldsymbol{\sigma}')$ is that of a sphere moving through a quiescent fluid with velocity $\boldsymbol{U}'$ and $\boldsymbol{\Omega}'$

$$\boldsymbol{u}'(\boldsymbol{r}) = \boldsymbol{U}' \cdot \boldsymbol{A} + \boldsymbol{\Omega}' \times \boldsymbol{b} \tag{54}$$

The hydrodynamic force density is equal and opposite to the electric force density for the primary flow, $\nabla \cdot \boldsymbol{\sigma} = -\nabla \cdot \boldsymbol{\sigma}_e$, and zero for the auxiliary flow, $\nabla \cdot \boldsymbol{\sigma}' = 0$. The surface integral over the outer surface $\mathcal{S}_\infty$ is zero since all fluid velocities and stresses decay sufficiently fast as $r \to \infty$. At the particle surface, the fluid velocity is equal to that of the particle. We can therefore write equation (53) as

$$\boldsymbol{U}' \cdot \int_{\mathcal{S}_p} (\boldsymbol{\sigma} \cdot \boldsymbol{n}) \mathrm{d}\mathcal{S} + \boldsymbol{\Omega}' \cdot \int_{\mathcal{S}_p} \boldsymbol{r} \times (\boldsymbol{\sigma} \cdot \boldsymbol{n}) \mathrm{d}\mathcal{S} - \boldsymbol{U} \cdot \boldsymbol{F}' - \boldsymbol{\Omega} \cdot \boldsymbol{L}' = \int_{\mathcal{V}} \boldsymbol{u}' \cdot (\nabla \cdot \boldsymbol{\sigma}_e) \mathrm{d}\mathcal{V} \tag{55}$$

where $\boldsymbol{F}' = -6\pi\eta a\boldsymbol{U}'$ and $\boldsymbol{L}' = -8\pi\eta a^3\boldsymbol{\Omega}'$ are the force and torque on the particle due to the auxiliary flow. The surface integrals of the hydrodynamic stress over the particle surface can be related to analogous integrals of the electric stress using equations (48) and (49), which state that the total force and torque on the particle surface is zero. Additionally, as

equation (55) is valid for any vectors $\boldsymbol{U}'$ and $\boldsymbol{\Omega}'$, we can separate it into two equations for translation and rotation, respectively,

$$6\pi\eta a\boldsymbol{U} = \int_{\mathcal{S}_p} (\boldsymbol{\sigma}_e \cdot \boldsymbol{n})\mathrm{d}\mathcal{S} + \int_{\mathcal{V}} \boldsymbol{A} \cdot (\nabla \cdot \boldsymbol{\sigma}_e)\mathrm{d}\mathcal{V} \tag{56}$$

$$8\pi\eta a^3\boldsymbol{\Omega} = \int_{\mathcal{S}_p} \boldsymbol{r} \times (\boldsymbol{\sigma}_e \cdot \boldsymbol{n})\mathrm{d}\mathcal{S} + \int_{\mathcal{V}} \boldsymbol{b} \times (\nabla \cdot \boldsymbol{\sigma}_e)\mathrm{d}\mathcal{V} \tag{57}$$

The integrals over the particle surface at $r = a$ can be related to analogous integrals over the bounding surface at $r \to \infty$ using the relations

$$\int_{\mathcal{S}_p} (\boldsymbol{\sigma}_e \cdot \boldsymbol{n})\mathrm{d}\mathcal{S} = -\int_{\mathcal{S}_\infty} (\boldsymbol{\sigma}_e \cdot \boldsymbol{n})\mathrm{d}\mathcal{S} - \int_{\mathcal{V}} (\nabla \cdot \boldsymbol{\sigma}_e)\mathrm{d}\mathcal{V} \tag{58}$$

$$\int_{\mathcal{S}_p} \boldsymbol{r} \times (\boldsymbol{\sigma}_e \cdot \boldsymbol{n})\mathrm{d}\mathcal{S} = -\int_{\mathcal{S}_\infty} \boldsymbol{r} \times (\boldsymbol{\sigma}_e \cdot \boldsymbol{n})\mathrm{d}\mathcal{S} - \int_{\mathcal{V}} \boldsymbol{r} \times (\nabla \cdot \boldsymbol{\sigma}_e)\mathrm{d}\mathcal{V} \tag{59}$$

Substituting this result into equations (56) and (57), we obtain

$$6\pi\eta a\boldsymbol{U} = -\int_{\mathcal{S}_\infty} (\boldsymbol{\sigma}_e \cdot \boldsymbol{n})\mathrm{d}\mathcal{S} + \int_{\mathcal{V}} (\boldsymbol{A} - \boldsymbol{I}) \cdot (\rho_e \boldsymbol{E})\mathrm{d}\mathcal{V} \tag{60}$$

$$8\pi\eta a^3\boldsymbol{\Omega} = -\int_{\mathcal{S}_\infty} \boldsymbol{r} \times (\boldsymbol{\sigma}_e \cdot \boldsymbol{n})\mathrm{d}\mathcal{S} + \int_{\mathcal{V}} (\boldsymbol{b} - \boldsymbol{r}) \times (\rho_e \boldsymbol{E})\mathrm{d}\mathcal{V} \tag{61}$$

where the electric force density is now expressed in terms of the charge density and the field: $\nabla \cdot \boldsymbol{\sigma}_e = \rho_e \boldsymbol{E}$. The surface integral in equation (60) is zero since the net charge of the particle and its double layer is zero; the surface integral in (61) is equal to the torque on the dipole $\boldsymbol{p}$ in the applied field $\boldsymbol{E}_o$.

Non-dimensionalization of the Model

To progress with the analysis, we non-dimensionalize the model outlined above using the following characteristic scales

$$\begin{aligned} [\boldsymbol{r}] &= a & [\Phi] &= k_B T/e \\ [\rho_e] &= 2ec_o & [\boldsymbol{u}] &= \varepsilon a(k_B T/ea)^2/\eta \\ [\rho_i] &= 2c_o & [p] &= \varepsilon(k_B T/ea)^2/\eta \end{aligned}$$

where $\rho_i = c_+ + c_-$ is the total ion density. We reformulate the governing equations and boundary conditions using these scales and discuss the various dimensionless parameters that emerge. For simplicity of notation, we use the same symbols for dimensional and dimensionless variables alike; the distinction should be clear from the context.

In dimensionless form, the ion transport problem for the ion density ρ_i, the charge density ρ_e, and the potential Φ can be written as

$$\nabla \cdot \boldsymbol{j}_i = \nabla \cdot (-\nabla\rho_i - \rho_e \nabla\Phi) = 0 \tag{62}$$

$$\nabla \cdot \boldsymbol{j}_e = \nabla \cdot (-\nabla\rho_e - \rho_i \nabla\Phi) = 0 \tag{63}$$

$$\nabla^2\Phi = -\beta^2 \rho_e \tag{64}$$

where $\beta = \kappa a$ is the ratio between the particle radius and the screening length, $\kappa^{-1} = (\varepsilon k_B T / 2e^2 c_o)^{1/2}$. Far from the particle, the boundary conditions become

$$\rho_i(\boldsymbol{r}) = 1 \quad \text{for} \quad r \to \infty \tag{65}$$

$$\rho_e(\boldsymbol{r}) = 0 \tag{66}$$

$$\Phi(\boldsymbol{r}) = -\boldsymbol{r} \cdot \boldsymbol{E}_o \tag{67}$$

where $\boldsymbol{E}_o$ is the applied field (now scaled by $k_B T/ea$). At the surface of the sphere, there is no flux of ions

$$\boldsymbol{n} \cdot \boldsymbol{j}_i(\boldsymbol{r}) = 0 \quad \text{for} \quad r = 1 \tag{68}$$

$$\boldsymbol{n} \cdot \boldsymbol{j}_e(\boldsymbol{r}) = 0 \tag{69}$$

The electric potential is subject to the following conditions on and off the metal patch

$$\Phi(\boldsymbol{r}) = \zeta + \boldsymbol{\gamma} \cdot \boldsymbol{E}_o \qquad \text{for } r = 1 \text{ on the patch} \tag{70}$$

$$-\boldsymbol{n} \cdot \nabla \Phi(\boldsymbol{r}) = \sigma \qquad \text{for } r = 1 \text{ off the patch} \tag{71}$$

where ζ describes the surface potential on the metal patch (scaled by $k_B T/e$), and σ describes the surface charge off the patch (scaled by $\varepsilon k_B T/ea$). The parameter $\boldsymbol{\gamma}$ is determined by the condition that

$$q_{\text{eq}} = \int_{\mathcal{S}_p} \boldsymbol{n} \cdot (-\nabla \Phi(\boldsymbol{r})) \mathrm{d}\mathcal{S} \tag{72}$$

where q_{eq} is the equilibrium charge on the particle surface (scaled by $\varepsilon a k_B T/e$).

The scaled model is characterized by four dimensionless parameters: the screening parameter β, the applied field $\boldsymbol{E}_o$, the zeta potential ζ, and the surface charge σ. In deionized water at 20°C, the screening length is $\kappa^{-1} = 1$ μm, which is comparable to the particle radius of $a = 1$ μm. The screening parameter is therefore estimated as $\beta = 1$ and increases with increasing ion concentration (e.g., due to dissolved CO_2). The applied field of 250 V/cm corresponds to a dimensionless field strength of $E_o = 1$. The zeta potential of the gold patch is estimated to be -25 mV, which corresponds to $\zeta = -1$. Based on the measured zeta potential of the bare particles (-40 mV), the surface charge parameter is estimated to be $\sigma = -3.2$. we make use of these parameter estimates in the numerical analysis below, while acknowledging that their true values are rather uncertain.

Perturbation Theory

To solve for the ICEP velocities $\boldsymbol{U}$ and $\boldsymbol{\Omega}$, we expand the ion density, the charge density, and the electric potential in powers of the applied field

$$\rho_i(\boldsymbol{r}) = \rho_i^{(0)}(\boldsymbol{r}) + E_o \rho_i^{(1)}(\boldsymbol{r}) + E_o^2 \rho_i^{(2)}(\boldsymbol{r}) + \ldots \tag{73}$$

$$\rho_e(\boldsymbol{r}) = \rho_e^{(0)}(\boldsymbol{r}) + E_o \rho_e^{(1)}(\boldsymbol{r}) + E_o^2 \rho_e^{(2)}(\boldsymbol{r}) + \ldots \tag{74}$$

$$\Phi(\boldsymbol{r}) = \Phi^{(0)}(\boldsymbol{r}) + E_o \Phi^{(1)}(\boldsymbol{r}) + E_o^2 \Phi^{(2)}(\boldsymbol{r}) + \ldots \tag{75}$$

We then substitute these expansions into the governing equations and boundary conditions, collecting like terms in E_o.

Zeroth Order, $O(E_o^0)$

At zeroth order in the applied field, we obtain the following description of the equilibrium double layer

$$\nabla \cdot \boldsymbol{j}_i^{(0)} = \nabla \cdot \left(-\nabla \rho_i^{(0)} - \rho_e^{(0)} \nabla \Phi^{(0)} \right) = 0 \tag{76}$$

$$\nabla \cdot \boldsymbol{j}_e^{(0)} = \nabla \cdot \left(-\nabla \rho_e^{(0)} - \rho_i^{(0)} \nabla \Phi^{(0)} \right) = 0 \tag{77}$$

$$\nabla^2 \Phi^{(0)} = -\beta^2 \rho_e^{(0)} \tag{78}$$

At equilibrium, the fluxes are everywhere zero, $\boldsymbol{j}_i^{(0)} = \boldsymbol{j}_e^{(0)} = 0$, and the ion concentrations are Boltzmann distributed such that $\rho_i^{(0)} = \cosh(\Phi^{(0)})$ and $\rho_e^{(0)} = -\sinh(\Phi^{(0)})$. The potential is governed by the Poisson-Boltzmann equation

$$\nabla^2 \Phi^{(0)} = \beta^2 \sinh(\Phi^{(0)}) \tag{79}$$

subject to boundary conditions

$$\Phi^{(0)}(\boldsymbol{r}) = 0 \qquad \text{for } r \to \infty \tag{80}$$

$$\Phi^{(0)}(\boldsymbol{r}) = \zeta \qquad \text{for } r = 1 \text{ on the patch} \tag{81}$$

$$-\boldsymbol{n} \cdot \nabla \Phi^{(0)}(\boldsymbol{r}) = \sigma \qquad \text{for } r = 1 \text{ off the patch} \tag{82}$$

First Order, $O(E_o)$

At first order in the applied field, we obtain the following description of the field-induced perturbation to the equilibrium double layer

$$\nabla \cdot \boldsymbol{j}_i^{(1)} = \nabla \cdot \left(-\nabla \rho_i^{(1)} - \rho_e^{(0)} \nabla \Phi^{(1)} - \rho_e^{(1)} \nabla \Phi^{(0)} \right) = 0 \tag{83}$$

$$\nabla \cdot \boldsymbol{j}_e^{(1)} = \nabla \cdot \left(-\nabla \rho_e^{(1)} - \rho_i^{(0)} \nabla \Phi^{(1)} - \rho_i^{(1)} \nabla \Phi^{(0)} \right) = 0 \tag{84}$$

$$\nabla^2 \Phi^{(1)} = -\beta^2 \rho_e^{(1)} \tag{85}$$

These governing equations are subject to following boundary conditions

$$\rho_i^{(1)}(\boldsymbol{r}) = 0 \qquad \text{for } r \to \infty \tag{86}$$

$$\rho_e^{(1)}(\boldsymbol{r}) = 0 \qquad \text{for } r \to \infty \tag{87}$$

$$\Phi^{(1)}(\boldsymbol{r}) = 0 \qquad \text{for } r \to \infty \tag{88}$$

$$\boldsymbol{n} \cdot \boldsymbol{j}_i^{(1)}(\boldsymbol{r}) = 0 \qquad \text{for } r = 1 \tag{89}$$

$$\boldsymbol{n} \cdot \boldsymbol{j}_e^{(1)}(\boldsymbol{r}) = 0 \qquad \text{for } r = 1 \tag{90}$$

$$\Phi^{(1)}(\boldsymbol{r}) = \boldsymbol{\gamma} \cdot \hat{\boldsymbol{E}}_o \qquad \text{for } r = 1 \text{ on the patch} \tag{91}$$

$$-\boldsymbol{n} \cdot \nabla \Phi^{(1)}(\boldsymbol{r}) = 0 \qquad \text{for } r = 1 \text{ off the patch} \tag{92}$$

The parameter $\boldsymbol{\gamma}$ is determined by the condition

$$0 = \int_{\mathcal{S}_p} \boldsymbol{n} \cdot (-\nabla \Phi^{(1)}(\boldsymbol{r})) \mathrm{d}\mathcal{S} \tag{93}$$

which states that the field does not alter the charge on the particle surface.

Second Order, $O(E_o^2)$

Finally, at second order in the applied field, we obtain the following description

$$\nabla \cdot \boldsymbol{j}_i^{(2)} = \nabla \cdot \left(-\nabla \rho_i^{(2)} - \rho_e^{(1)} \nabla \Phi^{(1)} - \rho_e^{(0)} \nabla \Phi^{(2)} - \rho_e^{(2)} \nabla \Phi^{(0)} \right) = 0 \tag{94}$$

$$\nabla \cdot \boldsymbol{j}_e^{(2)} = \nabla \cdot \left(-\nabla \rho_e^{(2)} - \rho_i^{(1)} \nabla \Phi^{(1)} - \rho_i^{(0)} \nabla \Phi^{(2)} - \rho_i^{(2)} \nabla \Phi^{(0)} \right) = 0 \tag{95}$$

$$\nabla^2 \Phi^{(2)} = -\beta^2 \rho_e^{(2)} \tag{96}$$

These governing equations are subject to following boundary conditions

$$\rho_i^{(2)}(\boldsymbol{r}) = 0 \qquad \text{for } r \to \infty \tag{97}$$

$$\rho_e^{(2)}(\boldsymbol{r}) = 0 \qquad \text{for } r \to \infty \tag{98}$$

$$\Phi^{(2)}(\boldsymbol{r}) = 0 \qquad \text{for } r \to \infty \tag{99}$$

$$\boldsymbol{n} \cdot \boldsymbol{j}_i^{(2)}(\boldsymbol{r}) = 0 \qquad \text{for } r = 1 \tag{100}$$

$$\boldsymbol{n} \cdot \boldsymbol{j}_e^{(2)}(\boldsymbol{r}) = 0 \qquad \text{for } r = 1 \tag{101}$$

$$\Phi^{(2)}(\boldsymbol{r}) = 0 \qquad \text{for } r = 1 \text{ on the patch} \tag{102}$$

$$-\boldsymbol{n} \cdot \nabla \Phi^{(2)}(\boldsymbol{r}) = 0 \qquad \text{for } r = 1 \text{ off the patch} \tag{103}$$

ICEP Velocity

Substituting the perturbation expansions into equations (50) and (51), the ICEP velocity $\boldsymbol{U}$ and $\boldsymbol{\Omega}$ are expressed as

$$\boldsymbol{U} = \frac{E_o^2}{6\pi} \int_{\mathcal{V}} (\boldsymbol{A} - \boldsymbol{I}) \cdot (\rho_e^{(1)} \boldsymbol{E}^{(1)} + \rho_e^{(0)} \boldsymbol{E}^{(2)} + \rho_e^{(2)} \boldsymbol{E}^{(0)}) \mathrm{d}\mathcal{V} \tag{104}$$

$$\boldsymbol{\Omega} = \frac{E_o^2}{8\pi} \left[(\boldsymbol{p}^{(1)} \times \hat{\boldsymbol{E}}_o) + \int_{\mathcal{V}} (\boldsymbol{b} - \boldsymbol{r}) \times (\rho_e^{(1)} \boldsymbol{E}^{(1)} + \rho_e^{(0)} \boldsymbol{E}^{(2)} + \rho_e^{(2)} \boldsymbol{E}^{(0)}) \mathrm{d}\mathcal{V} \right] \tag{105}$$

Note that the zeroth order terms in the force density can be absorbed into the pressure and do not contribute to the particle velocity. Similarly, the first order contributions to the particle velocity (i.e., the electrophoretic velocity) cancel for time oscillating fields with zero mean. The leading order contributions are second order in the applied field.

Numerical Results

The successive terms in the perturbation expansion are solved numerically using a commercial finite element solver (COMSOL v5.3) on a spherical domain of radius $R = 10$ centered on the particle. At successive orders, the electric potential at $r = R$ is approximated by the conditions

$$\Phi^{(0)}(\boldsymbol{r}) = 0 \quad \text{for} \quad r = R \tag{106}$$

$$\boldsymbol{r} \cdot \nabla \Phi^{(1)}(\boldsymbol{r}) + 2\Phi^{(1)}(\boldsymbol{r}) = -3\boldsymbol{r} \cdot \hat{\boldsymbol{E}}_o \tag{107}$$

$$\Phi^{(2)}(\boldsymbol{r}) = 0 \tag{108}$$

The zeroth order solution decays exponentially with distance from the particle; consequently, the approximate condition (106) above is quite accurate. The first order solution decays like an electric dipole in a uniform field; the approximate condition (107) introduces errors of order R^{-3} (that of the neglected quadrupole). The second order solution decays like an electric quadrupole; the approximate condition (108) is also accurate to order R^{-3}.

To compute the shape tensors $\boldsymbol{C}$ and $\boldsymbol{D}$, we compute the linear and angular velocity for several orientations of the applied field $\hat{\boldsymbol{E}}_o$. We then use linear regression to estimate the unknown parameters of the shape tensors. This approach is illustrated in Figure 9 for a patchy C_s particle with a deposition angle of 15°. For parameters $\beta = 1$, $\zeta = -1$, and $\sigma = -3.2$, the components of the rotation and translation tensors are computed to be

$$
\begin{array}{ll}
C'_{111} = -0.0233 & D'_{131} = -0.0278 \\
C'_{121} = -0.00557 & D'_{132} = -0.0544 \\
C'_{122} = 0.000120 & D'_{231} = 0.0380 \\
C'_{133} = -0.00335 & D'_{232} = 0.0221 \\
C'_{211} = -0.00174 & D'_{311} = 0.0561 \\
C'_{221} = -0.00711 & D'_{321} = 0.0158 \\
C'_{222} = -0.0162 & D'_{322} = -0.0442 \\
C'_{233} = -0.00686 & D'_{333} = -0.000194 \\
C'_{331} = -0.00987 & \\
C'_{332} = -0.00936 &
\end{array}
$$

In the computed particle trajectory, the particle aligns its mirror plane normal to the applied field and moves along circular orbits of radius $39a$. Assuming a particle radius of $a = 1$ μm and field strength of $E_o = 250$ V/cm, the predicted particle speed is 3.4 μm/s.

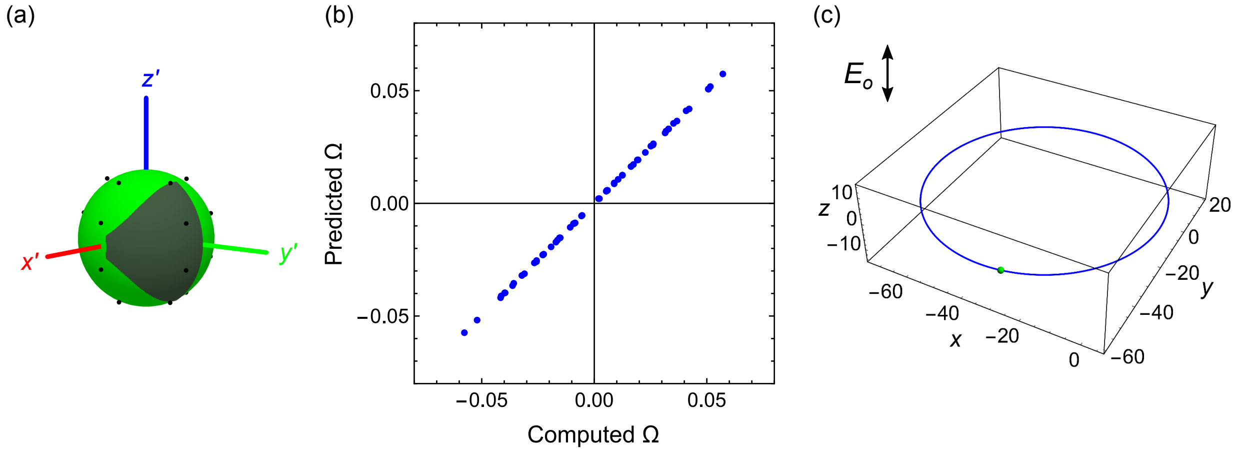

Supplementary Figure 9: (a) Model patchy particle based on line-of-sight deposition onto a close-packed monolayer with a deposition angle of 15° from horizontal. The black dots denote the different orientations of the applied field $\hat{\boldsymbol{E}}_o$ used in computing the shape tensors. (b) Angular velocities Ω predicted by the best fit rotation tensor $\boldsymbol{D}$ vs those computed numerically. Each point is one component of the velocity (x, y, z) for one orientation of the applied field (black dots in (a)). (c) Computed trajectory of the particle when the field is applied along the z-direction. The dimensionless parameters are $\beta = 1$, $\zeta = -1$, and $\sigma = -3.2$. The particle traces a circular path of radius $39a$.

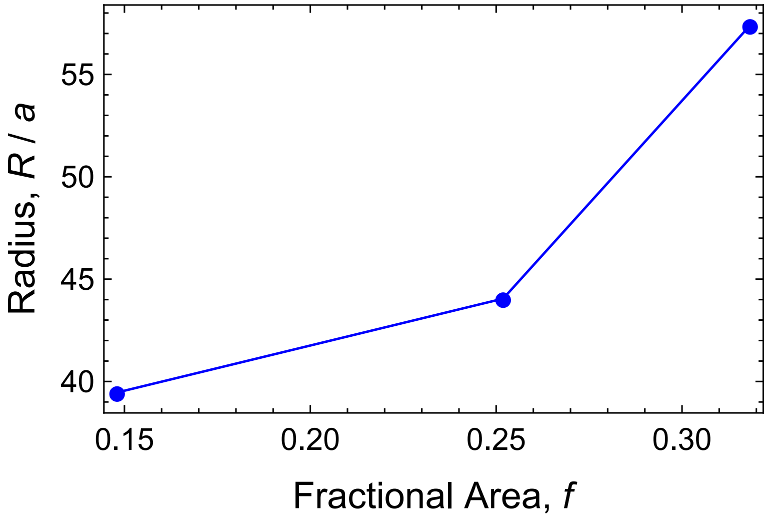

Supplementary Figure 10: Computed radius R of the circular trajectory (scaled by particle radius a) as a function of the fractional patch area f, corresponding to deposition angles $\phi = 15°, 30°, 40°$. The dimensionless parameters are $\beta = 1$, $\zeta = -1$, and $\sigma = -3.2$.

Supplementary Note 4: Role of Field Gradients

Spatial gradients in the applied field introduce forces due to dielectrophoresis (DEP) and alter the fluid flows that drive ICEP motions. For an ideally polarizable sphere in a uniform gradient, these two effects cancel one another resulting in no net motion of the sphere.[4] Here, we consider particle motion due to DEP as compared to ICEP motion in a uniform field, neglecting the effect of field gradients on the ICEP velocity. Field gradients can be neglected when motions due to DEP are negligible compared to ICEP motions. The DEP velocity of a patchy sphere is given by

$$\boldsymbol{U}_{\text{DEP}} = \frac{\boldsymbol{p} \cdot \nabla \boldsymbol{E}_o}{6\pi\eta a} \tag{109}$$

where $\boldsymbol{p} = \boldsymbol{\alpha} \cdot \boldsymbol{E}_o$ is the net dipole moment induced by the applied field, and $\boldsymbol{\alpha}$ is the polarizability tensor. From an order of magnitude perspective, such motions can be neglected provided that

$$\frac{U_{\text{DEP}}}{U_{\text{ICEP}}} \ll 1 \quad \text{or} \quad \frac{2}{3}\left(\frac{\alpha}{4\pi\varepsilon a^3}\right)\left(\frac{a\nabla E_o}{E_o}\right)\frac{1}{C} \ll 1 \tag{110}$$

The first term involving the polarizability is of order one as confirmed by numerical simulations on patchy spheres detailed above. The second term can be expressed as a/ℓ, where ℓ is a characteristic length for spatial variations in the applied field. The magnitude of the translation tensor C can be estimated from experiment to be $C = 0.008$, which agrees well with the numerical estimates detailed above. Therefore, DEP motions are negligible when $\ell \gg 100a$.

The geometry of the experimental cell is illustrated in Figure 11 below. Away from the electrodes, the applied field E is approximately uniform throughout the interior of the chamber. Within this region, the characteristic length ℓ is estimated to be $\ell \sim E_o/(\partial E_o/\partial x) \sim L$ where $L = 2$ mm is the distance between the electrodes. Therefore, we anticipate that the condition, $\ell \sim L \gg 100a$, is most certainly satisfied, such that field gradients are negligible except near the electrodes.

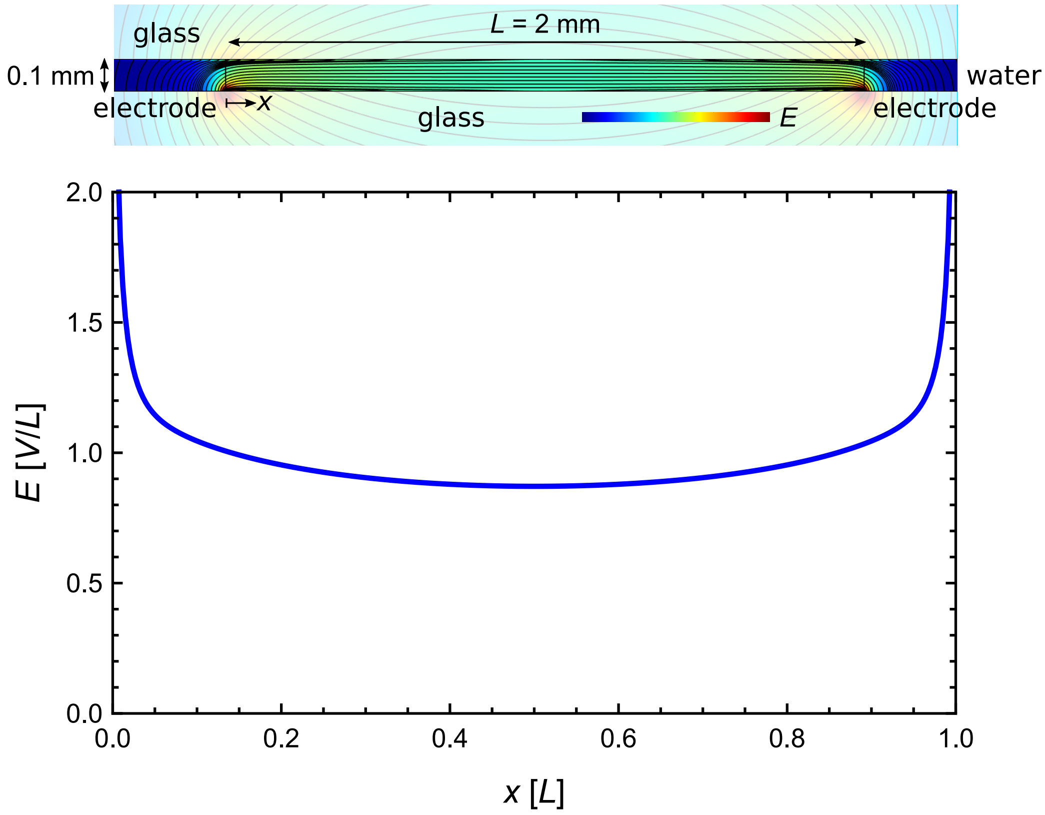

Supplementary Figure 11: Computed electric field distribution within the experimental chamber of height 100 μm. A voltage V is applied between two co-planar electrodes separated by a distance $L = 2$ mm. On time scales shorter than $L/D\kappa$, the aqueous electrolyte can be treated as a dielectric with permittivity ε, which is larger than the surrounding glass by a factor $\varepsilon/\varepsilon_g = 20$. The resulting electric field E within the electrolyte is approximately uniform within the interior of the chamber. The plot shows the computed magnitude of the electric field along the floor of the chamber.

Supplementary Note 5: Gravitational Effects

In a gravitational field $\boldsymbol{g}$, ICEP motions of a spherical particle are altered by gravitational forces and torques as

$$\boldsymbol{U} = \frac{\varepsilon a}{\eta}\boldsymbol{C} : \boldsymbol{E}_o\boldsymbol{E}_o + \frac{m\boldsymbol{g}}{6\pi\eta a} \tag{111}$$

$$\boldsymbol{\Omega} = \frac{\varepsilon}{\eta}\boldsymbol{D} : \boldsymbol{E}_o\boldsymbol{E}_o + \frac{m(\boldsymbol{x}_g - \boldsymbol{x}_h) \times \boldsymbol{g}}{8\pi\eta a^3} \tag{112}$$

where m is the buoyant mass of the particle, $\boldsymbol{x}_g$ is the gravitational center of the particle (i.e., the center of mass), and $\boldsymbol{x}_h$ hydrodynamic center of the particle (i.e., the geometric center of the sphere). In addition to the constant sedimentation velocity in equation (111), the gravitational field alters the orientation of the particle and thereby its ICEP motion in the electric field.

For the experimental conditions, however, we estimate that gravitational effects are largely negligible. In particular, we consider a 2 μm polystyrene sphere (density, 1.04 g/m^3) coated on one hemisphere by a 30 nm layer of gold (density, 19.3 g/m^3) and dispersed in water (density, 0.998 g/m^3 at 20°C). The buoyant mass of the particle is $m = 1.9 \times 10^{-15}$ kg, determined largely by the gold patch. Assuming line-of-sight deposition, the center of mass of the gold patch is displaced from the center of the sphere by a distance $2a/3$ in the direction of the patch. The sedimentation velocity of the particle in water (viscosity, 0.0010 Pa s) is estimated to be 1.0 μm/s; the rate of rotation due to gravity is 0.45 rad/s (26 deg/s). These values are smaller than the ICEP velocities observed in experiment (see Figure 2). Furthermore, we note that the trends in Figure 2 are not expected to hold when gravitational effects are significant.

Supplementary References

1. Brooks, A. M., Sabrina, S. & Bishop, K. J. M. Shape-directed dynamics of active colloids powered by induced-charge electrophoresis. *Proc. Natl. Acad. Sci.* **115,** E1090–E1099 (2018).
2. Yariv, E. Induced-charge electrophoresis of nonspherical particles. *Phys. Fluids* **17,** 051702 (2005).
3. Diebel, J. Representing attitude: Euler angles, unit quaternions, and rotation vectors. *Matrix* **58,** 1–35 (2006).
4. Squires, T. M. & Bazant, M. Z. Breaking symmetries in induced-charge electro-osmosis and electrophoresis. *J. Fluid Mech.* **560,** 65–101 (2006).
5. Bazant, M. Z. & Squires, T. M. Induced-charge electrokinetic phenomena: theory and microfluidic applications. *Phys. Rev. Lett.* **92,** 066101 (2004).
6. Stone, H. A. & Samuel, A. D. Propulsion of microorganisms by surface distortions. *Phys. Rev. Lett* **77,** 4102 (1996).
7. Kim, S. & Karrila, S. J. *Microhydrodynamics* (Dover, New York, 2005).
