# Supplementary material for: Directed propulsion of spherical particles along three dimensional helical trajectories
Source: Nat Commun. 2019 Jun 12;10:2575. doi: 10.1038/s41467-019-10579-1 (PMC6561940; doi:10.1038/s41467-019-10579-1)
Supplement: Supplementary file 3 — Description of Additional Supplementary Files [file 41467_2019_10579_MOESM3_ESM.pdf]

Description of Additional Supplementary Files

- *Supplementary Movie 1*:
- Directed motion of triangular patched spherical particles along helical trajectories of two distinct handedness and two directional propagation. The motion was ceased upon turning off the applied electric field.
- *Supplementary Movie 2:*
- Dynamic change in handedness of a particle in external electric field.
- *Supplementary Movie 3:*
- Bright field and fluorescence microscope video of a particle propelled along cylindrical helical trajectory.
- *Supplementary Movie 4:*
- Fluorescence microscope video showing the motion of particles with complex patch symmetry.
- *Supplementary Movie 5:*
- Bright field microscope video showing the dynamic transport of particles through a crosslinked matrix of irregular shaped pores.
